# Optimizing Poultry Nutrition to Combat *Salmonella*: Insights from the Literature

**DOI:** 10.3390/microorganisms12122612

**Published:** 2024-12-17

**Authors:** Muhammad Naeem, Dianna Bourassa

**Affiliations:** Auburn University, Auburn, AL 36849, USA; dvb0006@auburn.edu

**Keywords:** *Salmonella*, probiotics, prebiotics, organic acids, essential oils, poultry

## Abstract

*Salmonella* infections in poultry production are a significant and pervasive concern, posing substantial risks to both animal and human health. This comprehensive literature review examines the current body of research on the use of various nutritional manipulations as a promising strategy to effectively control and mitigate the prevalence of *Salmonella* in poultry. The review covers a range of dietary interventions, particularly the utilization of probiotics, prebiotics, organic acids, and phytochemicals, and thoroughly evaluates their efficacy in reducing *Salmonella* colonization within poultry flocks. Furthermore, the review delves into the potential underlying mechanisms of action for these nutritional approaches to control *Salmonella* and the far-reaching implications for overall food safety. By providing a thorough analysis of the existing literature, this review aims to elucidate the most promising nutritional strategies that can be employed to control *Salmonella* in poultry production, ultimately safeguarding animal welfare and public health.

## 1. Introduction

*Salmonella* is one of the leading bacterial causes of foodborne illnesses all over the world including the United States, frequently associated with poultry meat handling and consumption [1]. The Centers for Disease Control and Prevention estimate that there are approximately 1.2 million *Salmonella* infections in the US annually [1]. The European Centre for Disease Prevention and Control (ECDC) reports that in 2022, the EU/EEA recorded 65,967 confirmed cases of *Salmonellosis* in laboratories, corresponding to a notification rate of 15.5 cases per 100,000 people, while the UK government reported that England recorded 8125 cases of *Salmonella* in 2022, marking an increase from 2021 and the most frequently reported serovars were *Salmonella* Enteritidis and *Salmonella* Typhimurium.

Salmonellosis, the disease caused by *Salmonella* infection, is associated with major public health and economic concerns at both the local and global scales [2]. The prevalence of *Salmonella* in poultry production is a significant challenge, as it can lead to economic losses, food safety concerns, and potential public health risks [3]. *Salmonella*, a genus of Gram-negative bacteria, is a major public health concern worldwide due to its association with foodborne illnesses, particularly in relation to poultry products [4]. The escalating consumption of poultry and the prevalence of *Salmonella* in various poultry production and processing facilities have made the prevention and control of this pathogen a significant challenge for the industry. *Salmonella* is a global public health hazard, causing significant human illness and mortality. The disease in humans, known as *Salmonellosis*, is primarily associated with the consumption of contaminated poultry products, including eggs and chicken meat [2]. Furthermore, the increasing levels of antimicrobial resistance in *Salmonella* strains have exacerbated the problem, making effective treatment and control measures more challenging [1]. *Salmonella* Enterica serovars have been identified as one of the most important foodborne pathogens globally, with a wide range of animal hosts, including poultry, serving as vectors and reservoirs for the spread of these agents to human populations [5,6].

Conventional culture-based methods for *Salmonella* detection, while reliable, are time-consuming, typically taking 5–7 days to obtain a pure culture and serovar identification [7]. The development of molecular-based techniques, such as polymerase chain reaction, has revolutionized the field, enabling faster, more sensitive, and specific detection of *Salmonella* [8]. In addition to advancements in detection methods, the understanding of antimicrobial resistance patterns in *Salmonella* has become increasingly crucial. Animals are the most significant reservoirs of nontyphoidal *Salmonella* for humans, and resistance has been associated with antimicrobial use in animals, along with other management factors such as mixing of animals from different sources and transport [9]. *Salmonella* resistance to critically important antimicrobials, such as cephalosporins and fluoroquinolones, is a significant public health concern, as therapeutic options can be limited, particularly in certain patient populations [9]. The emergence of multidrug-resistant *Salmonella* Enterica serotype Newport in various geographic regions of the United States highlights the need for continued surveillance and the development of effective mitigation strategies [10].

The implementation of a complete ban on the use of growth-promoting antibiotics in animal feed in Europe in 2006 has further challenged poultry producers to improve production while reducing antimicrobial usage [5]. As a result, researchers have focused on exploring natural compounds and effective alternatives to prevent gastrointestinal diseases in poultry, including the use of nutraceuticals and phytonutrients [11]. These natural compounds have been investigated as potential solutions to the problem of *Salmonella* control in poultry, as the industry seeks to address public concerns about the appearance of antibiotic-resistant strains [12]. Furthermore, the emergence of antimicrobial-resistant *Salmonella* strains has become a serious problem, challenging the poultry industry to find alternative means of control [5]. Recognizing the importance of addressing this issue, researchers have explored various strategies to control *Salmonella* in poultry production. One such approach is the manipulation of poultry diets or nutritional interventions, which have shown promising results in reducing the prevalence and transmission of *Salmonella* in poultry flocks [1,2,4,5]. Traditionally, antibiotics have been used to control *Salmonella* in poultry; however, the emergence of antibiotic-resistant strains has led to a pressing need for alternative strategies [13].

The review aims to synthesize the current understanding of the role of nutritional interventions in controlling *Salmonella* in poultry. In this comprehensive literature review, we attempt to explore the potential of nutritional interventions as a strategy to mitigate the burden of *Salmonella* in poultry production.

## 2. Use of Probiotics to Combat *Salmonella* in Poultry

Probiotics are live microorganisms, including bacteria, fungi, and yeasts, that support the gut microbiota and contribute to a healthy digestive system. They enhance poultry growth performance and overall well-being and are increasingly used in poultry diets as a substitute for antibiotics [14,15]. Probiotics have been shown to inhibit the colonization and shedding of *Salmonella* in poultry, thereby reducing the risk of contamination and transmission to humans [2]. The mechanisms by which probiotics exert their anti-*Salmonella* effects include the production of antimicrobial compounds, competitive exclusion of the pathogen, and the modulation of the host’s immune response [1]. It has been reported in the literature that probiotics, particularly lactic acid bacteria (LAB) and *Saccharomyces* boulardii (SB), present promising options for reducing *Salmonella* colonization in broilers. A previous study [16] evaluated the inhibitory effects of LAB and SB against *Salmonella* Heidelberg (SH) in both in vitro and in vivo broiler models, examining their influence on faecal metabolites and cecal microbiome composition. In vitro testing showed strong inhibition of SH by specific probiotic strains, such as *Lactiplantibacillus* plantarum and *Lacticaseibacillus* acidophilus. In vivo analysis indicated that broilers provided with probiotics had significantly lower SH levels in cecal content compared to the positive control (PC) across all ages, demonstrating the probiotics’ protective role against SH colonization. Metagenomic analysis of cecal microbiota revealed predominant bacterial families and genera, showing shifts in microbiome composition influenced by age and probiotic use. Faecal metabolomics profiling also revealed changes in metabolite levels, suggesting reduced oxidative stress, decreased intestinal inflammation, and improved gut health status in birds supplemented with probiotics. These results emphasize the potential of probiotics to reduce SH colonization, improve broiler health, and decrease dependence on antibiotics. Similarly, Shao et al. [17] observed that *Enterococcus* faecium caused a significant decrease in *Salmonella* intestinal colonization and translocation, and improved gut health, and immune responses in broiler chickens.

Wu et al. [18] reported that probiotics promote the immune response of poultry birds and play an important role in reducing inflammation, thereby improving gut health. These researchers concluded that probiotic supplementation improves poultry’s growth rate and feed efficiency. In particular, they showed that probiotics, particularly microencapsulated probiotics, significantly reduce *Salmonella* Typhimurium load in broilers, promote growth performance, modulate cecal microflora, and decrease intestinal and liver inflammation by regulating pro-inflammatory and anti-inflammatory cytokines, offering a promising alternative to antibiotic growth promoters. A previous study [19] also showed that oral application of probiotics and prebiotics significantly improved the survival rates of the infected chicks with *Salmonella*. Notably, the *Bacillus* subtilis probiotic showed the most effective results in reducing the growth of *Salmonella* spp., particularly *Salmonella* Typhimurium and *Salmonella* Enteritidis. This indicates that probiotics and prebiotics can be beneficial in enhancing the survival of chickens challenged with virulent strains of *Salmonella*, suggesting their potential use in poultry farming practices to combat infections [19]. Some of the probiotics have also been reported to enhance the serum metabolites. For example, a study by Šefcová et al. [20] reported that probiotics, specifically *Lactobacillus* fermentum, improved intestinal health and enhanced serum IgM levels in chickens when they were infected with *Salmonella* Infantis. These probiotics alleviated the negative effects on intestinal architecture and enhanced immune response, thus suggesting the potential for reducing *Salmonella* colonization in poultry. Focusing on public health concerns, Limmaneevichitr [21] reported that probiotic supplementation plays a critical role in improving public health by decreasing the reliance on antibiotic growth promoters as probiotics supplementation showed potential in controlling *Salmonella*. However, the results of the supplementation of probiotics to poultry are not always consistent, a fact that can be attributed to the use, which appears to be due to the use of different strains and variability in efficacy. Hence challenges like strain selection and variability in the efficacy of the strains still need to be addressed in future research.

Recent research by Peng et al. [22] reported that *Lacticaseibacillus* rhamnosus significantly reduces *Salmonella* infection in poultry enhancing intestinal barrier integrity, improving gut microbiota diversity, and decreasing inflammation, thereby increasing survival rates, daily weight gain, and reducing apoptotic cells in chicks infected with *Salmonella* Typhimurium. Most of the research has been conducted on the *Lactobacillus* probiotics. However, limited research is available on a wide range of probiotics. The main probiotic strains commonly used are given in Table 1.

Specific probiotics can produce organic acids, hydrogen peroxide, and bacteriocins that directly inhibit the growth of *Salmonella*. Additionally, probiotics can competitively exclude *Salmonella* by occupying adhesion sites and nutrients in the intestinal tract, preventing the pathogen from establishing itself. Furthermore, probiotics have been observed to stimulate the host’s innate and adaptive immune responses, enhancing the bird’s ability to clear *Salmonella* infections. These multifaceted mechanisms make probiotics a promising approach for controlling *Salmonella* in poultry production, especially in the context of reducing the reliance on antibiotics and addressing the challenge of antimicrobial resistance.

## 3. Use of Prebiotics to Combat *Salmonella* in Poultry

In addition to probiotics, the use of prebiotics, which are non-digestible carbohydrates that selectively stimulate the growth and activity of beneficial gut microorganisms, has also been explored as a nutritional strategy to control *Salmonella* in poultry. Prebiotics, such as fructo-oligosaccharides, galacto-oligosaccharides, and inulin, have been shown to promote the growth of beneficial bacteria, such as Bifidobacterium and Lactobacillus, which can out-compete and inhibit the growth of *Salmonella* in the gastrointestinal tract [29]. Studies have demonstrated that the inclusion of prebiotics in poultry feed can reduce the colonization and shedding of *Salmonella* in broiler chickens and laying hens. The prebiotic-induced modulation of the gut microbiome can also enhance the bird’s immune response, further contributing to the control of *Salmonella* [30]. The mechanisms by which prebiotics can reduce *Salmonella* colonization include the production of short-chain fatty acids, which can inhibit the growth of pathogens, and the alteration of the gut microbiome, which can enhance the host’s immune response and increase resistance to *Salmonella* infections [30,31]. Richards et al. [31] supplemented the broilers with prebiotic galacto-oligosaccharides and observed enhanced *Salmonella* clearance in poultry by promoting beneficial gut microbiota, particularly increasing propionate and valerate levels, which inhibit *Salmonella* without triggering pro-inflammatory responses, thus improving gut health and reducing infection risk in broiler chickens. Tarabees et al. [32] evaluated the effect of probiotic, prebiotic and symbiotic and reported that prebiotic supplementation significantly improved immune response and down-regulated IL-6 and iNOS gene expressions in broilers infected with *Salmonella*. It also enhanced total IgY levels and positively affected haematological and oxidant-antioxidant biomarkers compared to the positive control group. In addition, prebiotics enhance the growth of beneficial bacteria, which can outcompete *Salmonella* for resources, thereby reducing its colonization [31]. Prebiotics are beneficial; however, their effectiveness can vary based on diet composition and individual animal responses, indicating a need for tailored approaches in poultry nutrition. Prebiotics commonly used for poultry are given in Table 2.

## 4. Use of Organic Acids to Combat *Salmonella* in Poultry

Another nutritional intervention that has been extensively studied is the use of organic acids, such as lactic acid, acetic acid, and butyric acid, as antimicrobial agents to control *Salmonella* in poultry. Organic acids have been shown to effectively inhibit the growth and survival of *Salmonella* in various poultry products, including meat and eggs. The antimicrobial activity of organic acids is attributed to their ability to disrupt the cell membrane, interfere with the intracellular pH, and inhibit essential metabolic processes in *Salmonella* cells. Organic acids can be added to poultry feed or drinking water, or applied as part of a decontamination treatment, to reduce the prevalence of *Salmonella* in poultry flocks and products [38]. Various studies highlight the antibacterial properties of different organic acids, demonstrating their potential as alternatives to antibiotics in poultry production.

Organic acids provide a low pH environment in the gut which is toxic for the pathogenic bacteria, and they enhance gut health by promoting beneficial bacteria, which indirectly reduces *Salmonella* loads [39]. Secondly, a blended approach may also be used in controlling *Salmonella* in poultry as using a blend of organic acids in feed and water has shown promise in controlling *Salmonella* Heidelberg in broilers, improving feed conversion rates and reducing pathogen incidence [40].

The results on the inclusion of organic acids and their effects vary between the studies conducted which may be due to differences between the organic acids used and their concentrations. For example, Lee et al. [41] revealed that plasma-activated organic acids inactivated approximately 6.37 Log colony forming units (CFUs)/mL of *Salmonella* Typhimurium in chicken cuts, demonstrating significant antibacterial effects and offering an effective method for controlling this pathogen in poultry without adversely affecting lipid oxidation. However, Reham et al. [42] found that acetic, citric, and fumaric acids effectively reduced *Salmonella* Typhimurium counts in chicken meat, with logarithmic reductions of up to 2.85 log CFU/g for fumaric acid at 2%, demonstrating their antibacterial efficacy against this pathogen. The mechanism of action of the organic acids involves lowering the pH in the gut which is not favourable for the pathogenic microbes. Costa et al. [43] explained that propionic acid effectively inhibits *Salmonella* Typhimurium growth, with minimum inhibitory concentrations of pH 4.5. Baaboua et al. [44] reported tartaric, citric, and acetic acids inhibited *Salmonella* Typhimurium at concentrations of 0.312%, 0.625%, and 0.512%, respectively. Lactic acid showed varying effectiveness, demonstrating the potential of organic acids to control *Salmonella* in poultry feed and improve food safety.

Organic acids supplementation also improves gut health by improving the microstructures of the gut and increasing the beneficial bacteria. Hu et al. [39] supplemented the broiler chickens with butyric acid and found that organic acids, such as butyric acid, effectively reduce *Salmonella* load in poultry by improving gut health, enhancing intestinal morphology, and increasing beneficial bacteria. Organic acids serve as a safe alternative to antibiotics, mitigating *Salmonella* Enteritidis infection in broiler chickens.

The mode of supplementation of organic acids may have an impact on the response of the birds as organic acids can be included in the diets and water too. However, there is no consistency in the results which can claim that inclusion in the diets or the water is the best. Sausen et al. [40] used a blend of organic acids through the water. The organic acid blend significantly reduced *Salmonella* Heidelberg incidence in broilers, showing numerical effectiveness in intestines and swabs. They found that treatment via water yielded the best results in feed conversion and live weight at various ages compared with the treatment via diets. However, Ricke et al. [45] explained that formic acid is effective in limiting *Salmonella* spp. in poultry by being added to diets, which helps reduce foodborne pathogens in feed and the gastrointestinal tract, potentially triggering complex responses in both *Salmonella* and the indigenous microbial community.

Research has found that the inclusion of lactic acid in poultry feed significantly reduced the shedding of *Salmonella* in broiler chickens, while another investigation demonstrated that the combination of lactic and acetic acids in drinking water effectively reduced *Salmonella* counts in the crop and ceca of layer hens. Similarly, the use of butyric acid as a feed additive has been shown to enhance the intestinal health of broilers and decrease the colonization of *Salmonella* in the gastrointestinal tract [46].

The effectiveness of organic acids in controlling *Salmonella* has made them an attractive alternative to traditional antimicrobial agents, particularly in light of the increasing prevalence of antibiotic-resistant *Salmonella* strains [38]. This suggests organic acids can be a viable alternative to antibiotics in poultry production. However, organic acids are effective in controlling *Salmonella*, and their application must be carefully managed to avoid potential sensory impacts on meat quality and ensure consumer acceptance.

## 5. Use of Phytochemicals to Combat *Salmonella* in Poultry

Phytochemicals have shown great potential in controlling *Salmonella* in poultry production. These plant-derived bioactive compounds, such as essential oils, phenolic compounds, and saponins, possess potent antimicrobial properties that can inhibit the growth and survival of *Salmonella* [47]. The antimicrobial mechanisms of phytochemicals include disrupting the pathogen’s cell membrane, interfering with intracellular processes, and enhancing the host’s immune response. Ongoing research is exploring the optimal use of phytochemicals, either alone or in combination with other nutritional interventions, to develop comprehensive strategies that leverage the synergistic effects of these compounds and other nutritional compounds or additive production [48]. This approach aims to provide a more robust and sustainable solution for managing *Salmonella* in the poultry industry.

One group of phytochemicals that have received significant attention are essential oils, which are volatile aromatic compounds extracted from various plant sources. Essential oils have been shown to possess potent antimicrobial properties against a wide range of bacteria, including *Salmonella* [49]. For example, studies have demonstrated that the incorporation of essential oils from oregano, thyme, or cinnamon into poultry feed or drinking water can significantly reduce the shedding and colonization of *Salmonella* in broiler chickens and laying hens [50]. The antimicrobial activity of essential oils is attributed to their ability to disrupt the cell membrane, interfere with cellular respiration, and inhibit the production of virulence factors in *Salmonella* [51]. Specifically, the active compounds in essential oils, such as thymol, carvacrol, and cinnamaldehyde, have been found to be particularly effective in disrupting the cell membrane of *Salmonella*, leading to the leakage of cellular contents and ultimately cell death [52]. Additionally, these compounds can interfere with the electron transport chain and ATP production, thereby impairing the cellular respiration of *Salmonella* [53]. Furthermore, essential oils have been shown to inhibit the expression of genes responsible for the production of virulence factors, such as flagella and fimbriae, which are crucial for the attachment and invasion of *Salmonella* into host cells. This multifaceted antimicrobial activity of essential oils makes them a promising intervention strategy for controlling *Salmonella* in poultry production.

The antimicrobial properties of essential oils against pathogenic microorganisms arise from multiple mechanisms. These include altering the intestinal lumen’s pH, impeding microbial adhesion to epithelial surfaces, reducing toxin production, inhibiting microbial proliferation, and preventing biofilm formation [54]. The hydrophobic nature of essential oils enables them to interact with microbial cell membranes, disrupting their structure. Additionally, essential oils partition into the lipids of bacterial cell walls and mitochondria, leading to division, structural compromise, and increased permeability [54,55]. However, essential oils may also influence beneficial gut microorganisms. For instance, in vitro studies show that oregano essential oil can suppress certain *Lactobacillus* species [55]. Since the effects of essential oils on beneficial microorganisms are not yet fully understood, ongoing research aims to clarify their mechanisms of action.

Santos et al. [56] reported that phytochemicals from *Cinnamomum amoenum* extracts demonstrated antimicrobial activity against various *Salmonella* serotypes in poultry, with ethanolic and ethyl acetate extracts showing significant inhibitory effects, suggesting their potential as sustainable alternatives to synthetic antimicrobials in poultry farming, whereas Thomas et al. [57] evaluated *Citrus limon* and *Bambusa polymorpha* extracts, achieving 3.95 and 1.88 log reductions in *Salmonella* Typhimurium in ground pork, respectively, indicating the potential effectiveness of phytochemicals in controlling *Salmonella* in meat products through antimicrobial properties. Phenolic acids e.g., gallic and vanillic acid have also been used as alternatives to antibiotics to control *Salmonella* in poultry. Alvarado-Martinez et al. [58] supplied the chickens with gallic acid and vanillic acid. These authors found that gallic acid and vanillic acid significantly reduced *Salmonella* Typhimurium levels and that phenolic acids altered microbiota without harming beneficial bacteria, suggesting their potential as alternatives to conventional antibiotics for pathogen control in poultry.

Research has demonstrated the beneficial effects of polyphenolic compounds when they are used in combination to obtain the synergistic effect on the intestinal health of the birds. Polyphenolic compounds, such as carvacrol and thymol, have been identified as effective natural feed additives that can enhance gut health and control antibiotic-resistant bacteria in poultry [59]. Al-Mnaser et al. [59] explained that phytochemicals, particularly tannins, inhibit *Salmonella* growth by acting as anti-quorum sensing and anti-virulence agents, destabilizing membranes, and chelating essential iron, thereby reducing avian diseases and the transmission of zoonotic pathogens in poultry.

A mixture of phytobiotics, including thymol and menthol, demonstrated strong antibacterial activity against *Salmonella* serovars, suggesting their potential for practical application in poultry farming [60], which may help control *Salmonellosis* and reduce antibiotic resistance in these bacteria. The researchers [60] found phytobiotics mixture effective against *Salmonella* Enteritidis, Typhimurium, and Kentucky. They reported minimum inhibitory concentration and minimum bactericidal concentration were 1:256. Thymol, carvacrol, and cinnamaldehyde effectively inhibit *Salmonella* Enteritidis in poultry, reducing microbial reproduction, pH, moisture content, and spoilage. These phytochemicals enhance chicken quality and shelf life, providing a natural alternative to traditional antibiotics in poultry management [61].

While phytochemicals show promise in controlling *Salmonella*, further research is necessary to fully understand their mechanisms and optimize their use in poultry production systems. By leveraging the complementary mechanisms of action of these different nutritional components, research is warranted to develop more effective and sustainable strategies to address the persistent challenge of *Salmonella* contamination in the poultry supply chain. Additionally, the potential for developing resistance to these natural agents should be considered.

## 6. Effect of Phytochemicals on Immune Response in Poultry

Beyond their direct antimicrobial effects, phytochemicals have also been shown to modulate the host’s immune response, potentially enhancing the bird’s resistance to *Salmonella* infections [47]. Certain phytochemicals, such as polyphenols and saponins, have been observed to stimulate the production of proinflammatory cytokines, increase the activity of macrophages and natural killer cells, and enhance the production of antibodies, all of which can contribute to a more effective immune response against *Salmonella* [62,63]. These phytochemicals can interact with the host’s immune system in multiple ways, including upregulating the expression of pattern recognition receptors, activating signalling pathways that promote the differentiation and proliferation of immune cells, and increasing the production of antimicrobial peptides. By modulating the host’s innate and adaptive immune responses, phytochemicals may provide an additional layer of protection against *Salmonella* infections, complementing their direct antimicrobial effects [64]. Furthermore, the modulation of the host’s immune response by phytochemicals can potentially lead to a more robust and sustained defence against *Salmonella*, reducing the likelihood of persistent or recurrent infections. Additionally, the enhancement of the host’s immune defence may help to mitigate the development of antimicrobial resistance, as the pathogen will face multiple barriers to successful colonization and infection [65].

## 7. Synergistic Effects of Nutritional Interventions for *Salmonella* Control

The available evidence indicates that the combined application of multiple nutritional strategies, including probiotics, prebiotics, organic acids, and phytochemicals, may provide a more comprehensive and effective approach to managing *Salmonella* in poultry production [66]. The synergistic interactions between these interventions can result in a more significant reduction in *Salmonella* colonization and shedding, as well as an improved immune response in the host. Some of the studies have reported the combined positive effect of probiotics and prebiotics on the reduction of *Salmonella*. Tarabees et al. [32] used a mixture of probiotics and prebiotics for the *Salmonella*-challenged birds and observed a significant decrease in *Salmonella* counts in broilers challenged with S. enteritidis and S. Typhimurium, enhancing growth performance and increasing *Lactobacillus* and *Enterococcus* populations in the caeca compared to the untreated challenge group.

This integrated approach allows for the targeting of *Salmonella* through various mechanisms, such as the inhibition of pathogen growth, the modulation of the gut microbiome, and the enhancement of the host’s immune defence [66]. Enan et al. [19] supplemented the birds with a combination of probiotics and prebiotics, such as Levoxyl, and reported significantly improved survival rates in infected poultry by enhancing immune responses and reducing *Salmonella* spp. growth. This strategy serves as an effective nutritional intervention to inhibit pathogens and promote overall poultry health and performance. These researchers found that the oral application of probiotics and prebiotics significantly improved the survival rates of the infected chicks compared with in-feed application. Notably, the B. subtilis probiotic showed the most effective results in reducing the growth of *Salmonella* spp., particularly S. Typhimurium and S. enteritidis. This appears that probiotics and prebiotics together can also be beneficial in enhancing the survival of chickens challenged with virulent strains of *Salmonella*, suggesting their potential use in poultry farming practices to combat infections. Not only in broilers but also in layer pullets, the combination of probiotics and prebiotics has been reported to bring beneficial effects on poultry. Kimminau et al. [67] used layer pullets in their research to investigate the combined effect of probiotics and prebiotics to reduce *Salmonella* infection. They found that the combination of prebiotics and probiotics significantly reduced *Salmonella* Enteritidis shedding in layer pullets, with lower *Salmonella* Enteritidis counts observed in treated birds compared to controls, indicating a beneficial effect of prebiotics on *Salmonella* infection.

Tarabees et al. [32] investigated the effects of probiotic, prebiotic, and synbiotic supplements on various health parameters in broilers infected with *Salmonella* to evaluate how these supplements influence immune response, gene expression related to inflammation, haematology, and oxidant-antioxidant biomarkers in broilers infected with *Salmonella* Enteritidis and *Salmonella* Typhimurium. They found that probiotic, prebiotic, and synbiotic supplements significantly increased total IgY levels in the sera of infected birds compared to the positive control group. These supplements also down-regulated the expression of pro-inflammatory markers like IL-6 and iNOS while up-regulating the anti-inflammatory marker IL-10 in the caeca of infected birds. Additionally, the treatments improved haematological parameters and oxidant-antioxidant biomarkers affected by *Salmonella* infection. By leveraging the complementary effects of these different nutritional interventions, poultry producers can develop a more robust and sustainable strategy to control *Salmonella* and ensure the safety and quality of poultry products.

## 8. Challenges and Future Directions

With the advancement of the discovery of new serotypes, for example, *Salmonella* Enterica serotype Newport, further research is required to elucidate the complex interplay between antimicrobial use in animal production, the emergence of resistance, and the potential for transmission to humans through the food chain and other environmental pathways [9]. The detection and characterization of *Salmonella*, as well as the understanding of antimicrobial resistance patterns, are critical for public health and food safety. Recent advancements in rapid detection methods and the exploration of non-antibiotic strategies for *Salmonella* control in poultry offer promising avenues for addressing this persistent challenge [68]. However, looking ahead, the continued development of novel detection technologies, such as biosensors and microfluidic devices, holds the potential to further improve the speed, sensitivity, and specificity of *Salmonella* identification [7]. Additionally, the implementation of a One Health approach, which recognizes the interconnectedness of human, animal, and environmental health, is crucial for the effective management of antimicrobial resistance in *Salmonella* and other foodborne pathogens [9]. Another non-antibiotic strategy gaining traction is the use of bacteriophages, or phages, to control *Salmonella* in poultry. Phage therapy has been explored as a promising alternative to traditional antibiotics, with the potential to target specific *Salmonella* strains without disrupting the gut microbiome [68].

While the use of nutritional interventions, such as probiotics, prebiotics, organic acids, and phytochemicals, holds promise for controlling *Salmonella* in poultry, there are some limitations and challenges that need to be addressed. Firstly, the efficacy of these interventions can be influenced by various factors, including the specific strain of *Salmonella*, the poultry production system, the age and health status of the birds, and the interaction with other dietary components [46,48,69,70]. Secondly, the development of antimicrobial resistance in *Salmonella* is a growing concern, and the overuse or improper application of these interventions could potentially contribute to the emergence of resistant strains [38]. However, the feasibility and commercial application of phytogenic substances are complicated by various challenges, including bioavailability, absorption rate, quality inconsistencies, public acceptance, and the development of cost-effective delivery methods [71]. Furthermore, the integration of these nutritional strategies into commercial poultry production requires careful consideration of factors such as cost-effectiveness, ease of implementation, and regulatory approval [38,46,48,66]. The phytochemical being used in the poultry industry may need validation of antimicrobial use in poultry *Salmonella* control. Additionally, the long-term effects of phytochemicals on human health are unclear and need more studies on phytochemical mechanisms. Finally, the economic feasibility and regulatory approval of these interventions must be carefully evaluated to ensure their widespread adoption in the poultry industry.

## 9. Conclusions

In conclusion, the literature review highlights the potential of nutritional interventions, including probiotics, prebiotics, organic acids, and phytochemicals, to effectively control *Salmonella* in poultry production. The antimicrobial and immunomodulatory properties of these interventions can work synergistically to reduce the shedding and colonization of *Salmonella* in broiler chickens and laying hens. While the available evidence is promising, the successful implementation of these strategies will require addressing the limitations and challenges, such as the influence of various factors on efficacy, the risk of antimicrobial resistance, and the economic and regulatory considerations. By developing a comprehensive and integrated approach to *Salmonella* control, the poultry industry can enhance the safety and quality of poultry products, while also promoting the sustainable and responsible use of antimicrobials.

## Figures and Tables

**Table 1:** Commonly used probiotics in poultry.

Probiotics	Effects	References
*Lactobacillus*	*Lactobacillus plantarum* and *Lactobacillus reuteri* are noted for their high survivability in gastrointestinal conditions and antimicrobial properties against pathogens like *Salmonella.*	[23,24]
*Bifidobacterium*	*Bifidobacterium longum* has shown significant attachment to the intestinal mucosa and inhibition of pathogenic microbes.	[23]
*Enterococcus*	*Enterococcus durans* has been characterized for its potential as a probiotic candidate, demonstrating resistance to gastrointestinal conditions.	[25]
*Saccharomyces*	*Saccharomyces boulardii* is recognized for its ability to survive under various pH levels and bile concentrations.	[23]
*Bacillus*	*Bacillus* strains are commonly included in poultry diets for their growth-promoting effects.	[26]
	*Bacillus amyloliquefaciens* are root-colonizing biocontrol bacteria utilized to combat plant root pathogens in agriculture, aquaculture, and hydroponics. They also promote gut health and improve growth performance.	[27]
	*Bacillus subtilis* are found in the soil and the gastrointestinal tract of ruminants and humans, play a role in enhancing laying performance and help the immune system and gut health.	[27]
	*Streptococcus faecium* bacteria inhabit the gastrointestinal tracts of humans and other mammals and improve immune functions.	[28]

**Table 2:** Commonly used prebiotics in poultry.

Prebiotics	Effects	References
Oligosaccharides	Fructo-oligosaccharides (FOS): Derived from plants, they stimulate beneficial bacteria in the gut.	[33]
	Xylo-oligosaccharides (XOS): Xylo-oligosaccharides (XOS) are known to have a prebiotic effect that supports the growth of a healthy microbiota, making them one of the most widely used oligosaccharides in the poultry industry.	[34]
	Galacto-oligosaccharides (GOS): Known for their ability to enhance gut health.	[33]
	Mannooligosaccharides (MOS): Sourced from yeast, they support the growth of beneficial gut flora.	[35]
Inulin	A type of fructan that promotes the growth of beneficial bacteria and improves nutrient absorption.	[35,36]
Lactulose	A synthetic disaccharide that enhances the growth of lactobacilli and bifidobacteria, improving gut health.	[35]
Other	Psyllium seeds and apple fibre have also shown prebiotic properties.	[37]

## Data Availability

Not applicable.
